# Circular Permutation Prediction Reveals a Viable Backbone Disconnection for Split Proteins: An Approach in Identifying a New Functional Split Intein

**DOI:** 10.1371/journal.pone.0043820

**Published:** 2012-08-24

**Authors:** Yun-Tzai Lee, Tz-Hsiang Su, Wei-Cheng Lo, Ping-Chiang Lyu, Shih-Che Sue

**Affiliations:** 1 Institute of Bioinformatics and Structural Biology, National Tsing Hua University, Hsinchu, Taiwan; 2 Department of Life Science, National Tsing Hua University, Hsinchu, Taiwan; 3 Department of Medical Science, National Tsing Hua University, Hsinchu, Taiwan; 4 Institute of Bioinformatics and Structural Biology, National Chiao Tung University, Hsinchu, Taiwan; King’s College, London, United Kingdom

## Abstract

Split-protein systems have emerged as a powerful tool for detecting biomolecular interactions and reporting biological reactions. However, reliable methods for identifying viable split sites are still unavailable. In this study, we demonstrated the feasibility that valid circular permutation (CP) sites in proteins have the potential to act as split sites and that CP prediction can be used to search for internal permissive sites for creating new split proteins. Using a protein ligase, intein, as a model, CP predictor facilitated the creation of circular permutants in which backbone opening imposes the least detrimental effects on intein folding. We screened a series of predicted intein CPs and identified stable and native-fold CPs. When the valid CP sites were introduced as split sites, there was a reduction in folding enthalpy caused by the new backbone opening; however, the coincident loss in entropy was sufficient to be compensated, yielding a favorable free energy for self-association. Since split intein is exploited in protein semi-synthesis, we tested the related protein *trans*-splicing (PTS) activities of the corresponding split inteins. Notably, a novel functional split intein composed of the N-terminal 36 residues combined with the remaining C-terminal fragment was identified. Its PTS activity was shown to be better than current reported two-piece intein with a short N-terminal segment. Thus, the incorporation of *in silico* CP prediction facilitated the design of split intein as well as circular permutants.

## Introduction

In the protein-engineering field, it is well known that manipulating individual residues can change the non-covalent interactions within a protein structural scaffold. Non-covalent contacts support protein structure and folding. However, the covalent connectivity provided by peptide bonds also restricts protein conformation because of steric hindrance and limited energy loss [1], [2]. Because the protein backbone connectivity is so restrictive, it acts as a critical factor in determining protein structure [3]. Circular permutation (CP) is an emerging method designed to introduce changes into protein sequences and thus protein structure [1]. CP is a backbone rearrangement in which the original amino- and carboxyl-termini in a protein sequence are connected and new termini internally relocated [1], [4]. Despite this rearrangement, many CPs retain their native structure and conserved function or enzymatic activity [1], [2]. Therefore, CP constitutes an alternative to protein engineering using site-directed mutagenesis [5], [6], [7]. A substantial number of natural examples of CP have been reported [4], [8] since the first identification of naturally occurring CP in plant lectins [9]. Examining the influences of CP on the structures and functions, the outcomes have indicated that if the proper CP site is used, the overall protein structure and biological function can be maintained [2], [10].

With the aim of making CP a practical method for protein engineering, we developed an efficient program, CPSARST (Circular Permutation Search Aided by Ramachandran Sequential Transformation), to search target protein for CPs in databases [11]. CPSARST translates the amino acid sequence into a text string based on the distribution of the backbone dihedral angle of each residue on a Ramachandran map [12]. The linear-coding string simplifies structural alignment; thus, protein CP searching is very efficient. Therefore, we performed a large-scale search for CP-related proteins in the Protein Data Bank (PDB) and created the first CP database, CPDB [13]. Surprisingly, more than 10% of the protein structures in the PDB contained CP relationships. This result confirmed the genetic importance of CP in protein evolution. Furthermore, the assessments allowed us to develop a CP predictor, CPred [14], [15]. This program is designed to rank possible CP sites in a given protein based on structural coordinates and output scores for each residue, thus predicting which CPs will fold properly. Using this program, we have successfully predicted the folding of both naturally occurring and artificially designed CPs [14], [16], demonstrating the superior abilities of the CPred program.

To extend the application of this program, we tested the hypotheses that a valid CP site can be used as a split site in a protein-engineering scheme and that the two-fragment protein generated in this process will fold properly and exert its biological function. When engineering such a structural arrangement, the structural constrains are relaxed at different degree through backbone cleavage. Therefore, the degree of protein folding is quite variable, ranging from completely folded to nearly disordered [3]. We speculate that valid CP sites might be great candidates for split sites because the new opening can be tolerated by the entire structural scaffold. Thus, detrimental effects on protein folding can often be avoided.

We used a protein ligase, intein, as a model to evaluate the idea. Intein functions in protein splicing [17]. It can covalently ligate two flanking protein sequences (extein), which are linked to the intein amino- and carboxyl-termini, respectively [17], [18]. In practice, the intein polypeptide chain could be split into two pieces [17], [18]. The functional two-piece intein should be able to spontaneously constitute the native intein fold and retain protein ligase activity, thus linking the two flanking exteins and catalyzing protein *trans*-splicing (PTS) [18]. We selected *Nostoc punctiforme* DnaE intein (NpuInt) based on its compact size (137 amino acids) and extraordinarily high protein splicing and *trans*-splicing activities [19], [20]. It has been used for other extending applications [21], [22], and its solution structure is available (PDB code: 2KEQ) [23], so the structural coordinate can be readily submitted to CPred.

To cross the gap of structural differences between the split intein and the single-chain CP intein, we first identified several stable intein CPs in which the openings had little effect on the overall intein structure. We then tested the conversion from CP site to split site and evaluated the effects on intein folding, stability and PTS activity.

## Results and Discussion

### Assessment of the Intein CP Site Prediction

**Figure 1:**
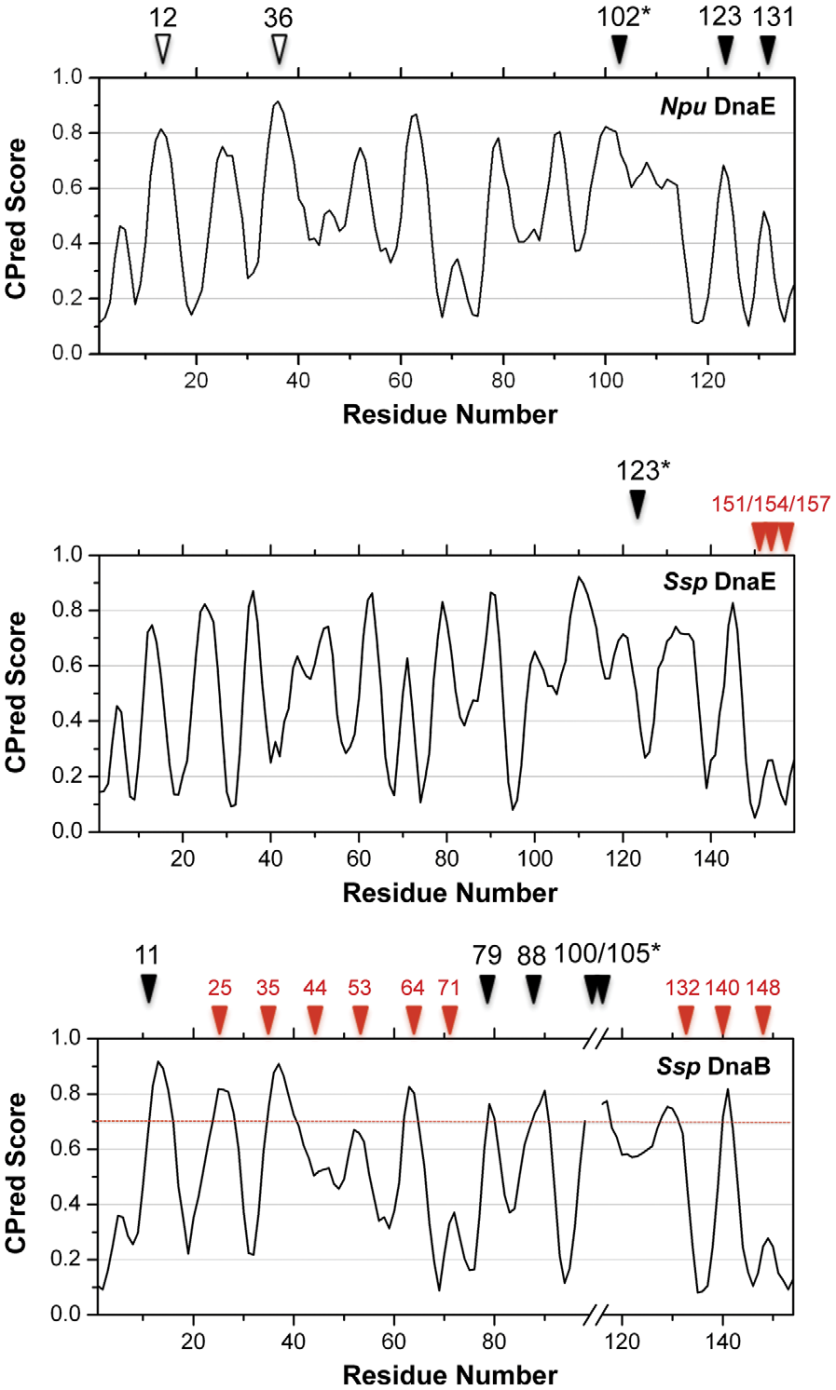
Predicted CP probabilities (CPred scores) for *Npu* DnaE (NpuInt), *Ssp* DnaE and *Ssp* DnaB inteins (*Npu: Nostoc punctiforme* and *Ssp: Synechchotcystis sp.*) plotted *versus* residue number. The coordinates of NpuInt (PDB code: 2KEQ) [23], *Ssp* DnaE (PDB code: 1ZD7) [25] and *Ssp* DnaB (PDB code: 1MI8) [24] were submitted to CPred to estimate the CP probability as a function of residue number. The locations of reported functional split sites (efficiency >50%) are indicated by closed black triangles, and asterisks indicate the naturally occurring split sites. The reported non-functional split sites in *Ssp* DnaE and *Ssp* DnaB are marked by closed red triangles. Two newly predicted *Npu* DnaE intein CP sites at residues 12 and 36 are indicated by open black triangles.

We analyzed three commonly used inteins [23], [24], [25] using CPred and correlated our results with previously reported split sites that support efficient PTS reactions [20], [23], [26] (Figure 1). CPred outputs a value between 0 and 1 for each residue, with scores closer to 1 corresponding to an increased likelihood for CP. We noticed that almost all of the functional split sites were at or near sites with local CPred score maximums. The only uncertainty is for the natural occurring split site 105 (Q105-L106) and engineered site 100 (E100-S101) in *Ssp* DnaB mini-intein; for this protein, the coordinates for residues 98–116 are missing in the X-ray structure (PDB code: 1MI8) [24]. Taking NpuInt as an example, the naturally occurring split site 102 (N102-I103) and the engineered split sites 123 (R123-D124) and 131 (N131-G132) [23] were well predicted by CPred, implying that this program can predict valid split sites as well as CP sites.

We also compared the functional and non-functional split sites in the same intein molecular frame. There were 13 split sites engineered in *Ssp* DnaB previously [26]. Together with the natural occurring split site 105, there were 5 functional versus 9 non-functional split sites (Figure 1). If we consider a threshold of CPred score  = 0.7, the five functional split sites are all with CPred scores higher than the value. Among the non-functional split sites, five of them are with lower CPred scores, and the other four split sites have high CPred score (>0.7). As analyzed in this case, if we count the split sites that meet the threshold (score >0.7), half of them (5 out of 9 sites) turned out to be functional. Thus, it confirms the proposition that CP sites with high CPred scores are competent to be protein backbone split sites. Moreover, we noticed the great correspondence between low CPred score (<0.5) and invalid backbone splitting. The predictor defines low CPred-score region where is not worthwhile to be tested as a split site. In final, we also gained similar observation in another case of *Ssp* DnaE (Figure 1).

Therefore, to identify new split sites in intein sequences, we firstly considered the sites with high CPred scores. Starting from the wide-type NpuInt, we chose potential sites based on three criteria. First, we chose positions with local CPred score maximums. Second, we chose positions with at least a moderate separation from the active site. Finally, we favored sites in the N-terminal portion because the reported functional split sites, including naturally occurring sites, are mostly distributed in the C-terminal half of intein [18]; thus, a new functional split intein with a split site in the N-terminal half has better potential for application. Under these conditions, we selected two sites, at residues 12 (E12-Y13) and 36 (N36-G37), both of which had CPred scores of higher than 0.8 and were located near the N-terminus. The naturally occurring split site at residue 102, which had a high CPred score, was also evaluated.

### Structural Similarity of CP Mutants of NpuInt

**Figure 2:**
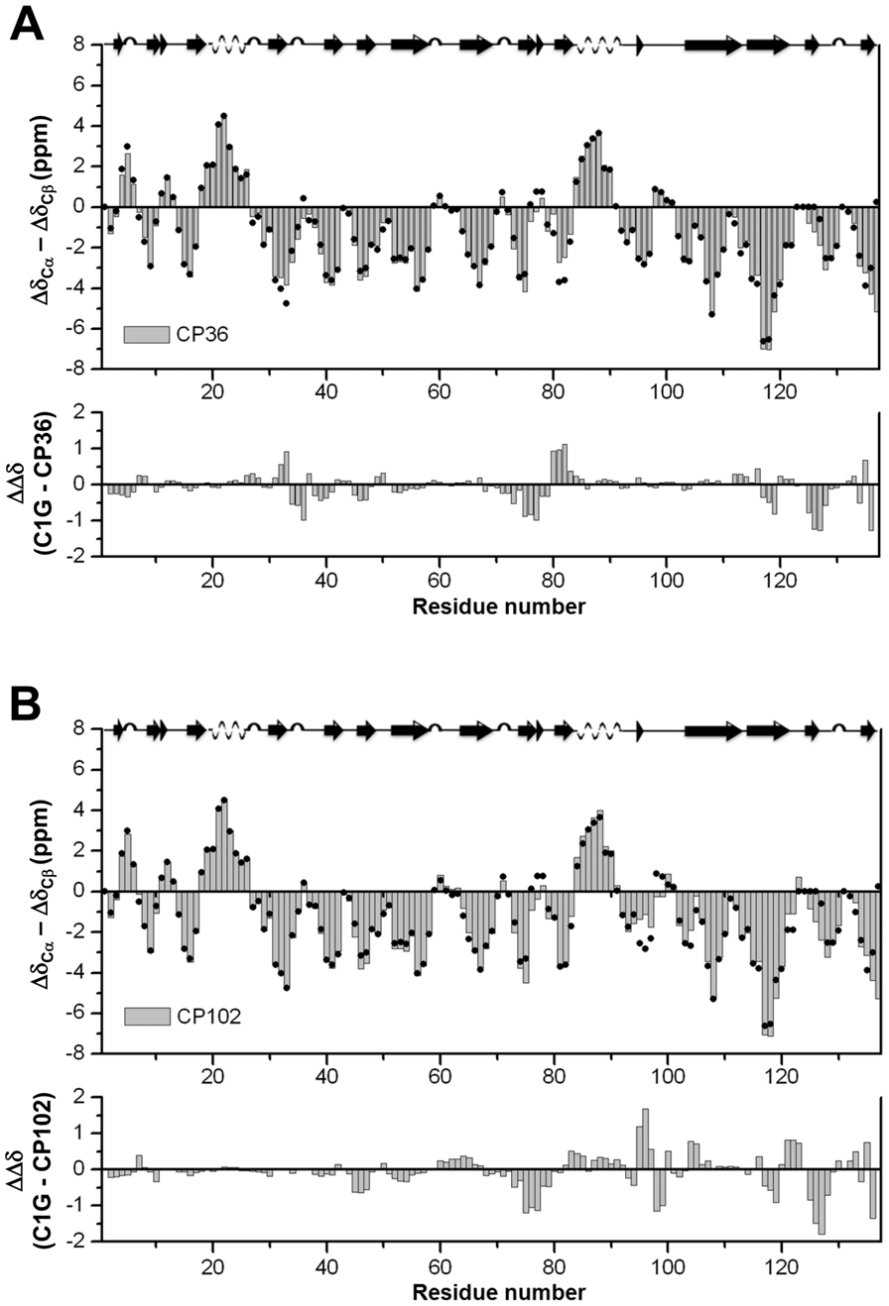
Secondary structure of the CP variants (A) CP36 and (B) CP102, evaluated according to the parameter Δδ_Cα_−Δδ_Cβ_. The chemical shift values for ^13^C_α_ and ^13^C_β_ of CP36 and CP102 were obtained, and Δδ_Cα_ and Δδ_Cβ_ were calculated from the differences between the experimental values and random coil values. The value of Δδ_Cα_−Δδ_Cβ_ for each residue represents the average of three consecutive residues, centered at the particular residue. The Δδ_Cα_−Δδ_Cβ_ value derived from native NpuInt C1G (indicated by closed circles) is overlaid onto the CP results for comparison. The corresponding secondary structure of C1G is depicted at the top. The difference (ΔΔδ) in (Δδ_Cα_−Δδ_Cβ_) between each CP variant and C1G was calculated and is indicated at the bottom of the figure.

**Figure 3:**
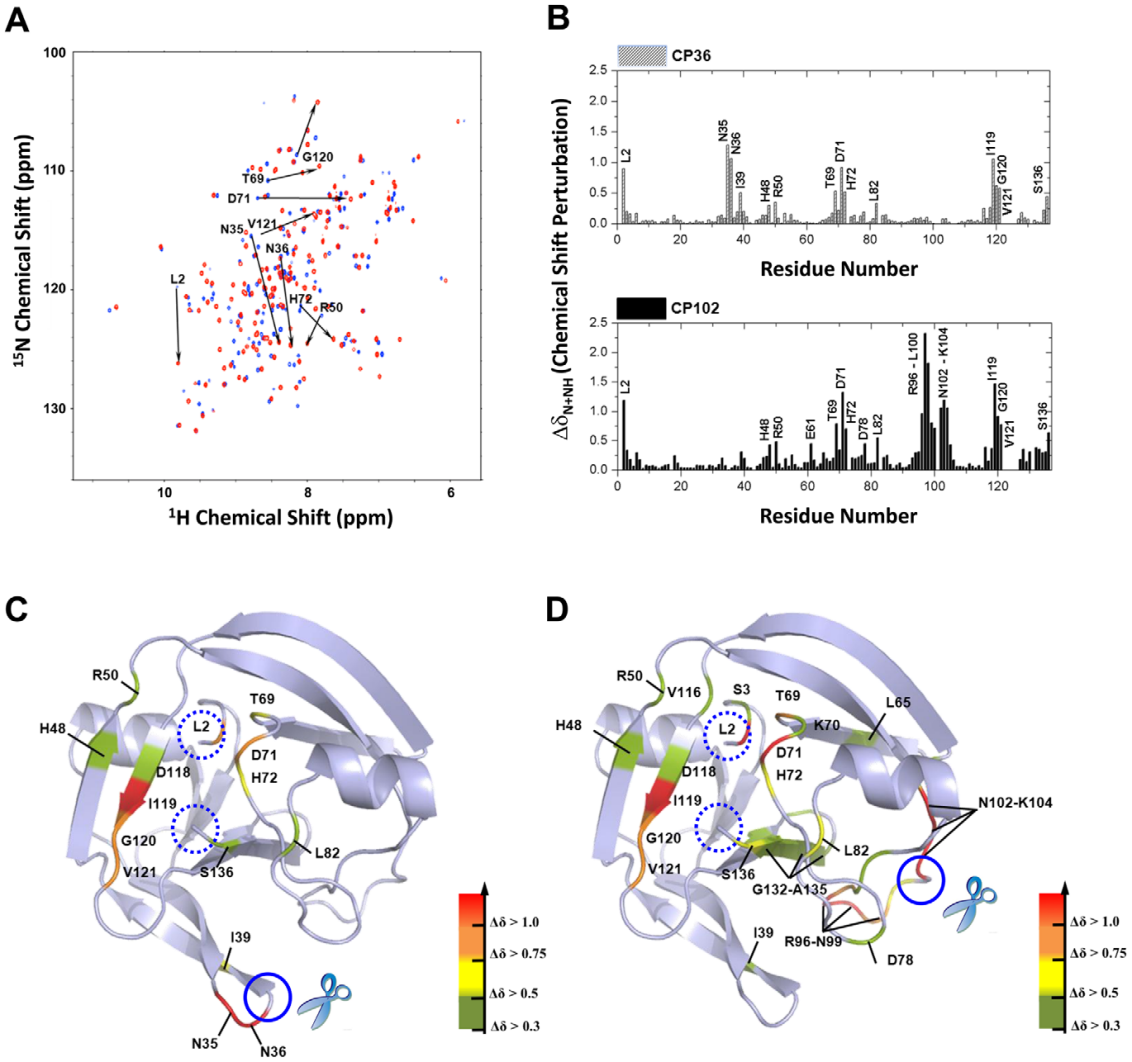
Tertiary structural comparison between CP variants and native NpuInt. (A) Superimposition of the ^1^H, ^15^N-HSQC spectra of native NpuInt C1G (blue) and CP36 (red). The spectra were taken at 25°C and 600 MHz. The residues with significant shifts are indicated. (B) Variation in the composite backbone NH and ^15^N chemical shift perturbation (Δδ_N+NH_) obtained from the spectra of CP36 and C1G (upper panel) and CP102 and C1G (lower panel), where Δδ_N+NH_ = [(Δδ_NH_^2^+Δδ_N_^2^/25)/2]^1/2^. Δδ_NH_ and Δδ_N_ were calculated from the differences for backbone NH and ^15^N, respectively. Spatial distribution of residues with significant Δδ_N+NH_ are mapped onto the NpuInt structure (PDB code: 2KEQ) [23], as indicated in different colors for (C) CP36 and (D) CP102. Dotted circles indicate the locations of the N- and C-terminal ends, and closed circles indicate the introduced CP sites.

To evaluate the NpuInt CPs, we prepared individual plasmids containing the desired CP coding sequences. A flexible three-residue linker connecting the original termini and new backbone disconnection was placed at the selected CP sites, resulting in the generation of CP12, CP36 and CP102. These NpuInt CPs were subjected to NMR analysis to evaluate the secondary and tertiary structural similarities. The chemical shifts of ^13^C_α_ and ^13^C_β_ are sensitive to the secondary structure of the backbone [27]. We used the parameter Δδ_Cα_−Δδ_Cβ_ to determine the α or β conformation of the backbone of each residue; a positive value for this parameter indicates an α-helix, and a negative value represents a β-strand. Comparing CP36 and CP102 to the native version of NpuInt, C1G, showed definitive correspondence (Figure 2). The differences observed were generally less than 2 ppm and were distributed in accordance with the distinct backbone linkages. Similarly, comparison of the NMR ^1^H-^15^N HSQC spectra of inteins CP36 and C1G (Figure 3A) revealed that the residues with significant chemical shift differences (L2, N35, D71, I119 and S136) were spatially or covalently close to the original N-terminus or the original C-terminus or the newly introduced opening (Figure 3B and C). This distribution indicated that these perturbations resulted from the distinct linkages in the polypeptide chain, not a conformational change. The idea was also supported by the excellent correspondence in the resonances of the residues in the rest of the protein. Therefore, we concluded that the secondary and tertiary structures of CP36 and C1G were very similar. A similar evaluation of CP102 yielded the same result (Figure 2B and Figure 3B and D). The complete comparison of ^1^H-^15^N HSQCs of CP12, CP36 and CP102 is included in Figure S1.

### Split Intein with a Backbone Disconnection at the Valid CP Site

**Figure 4:**
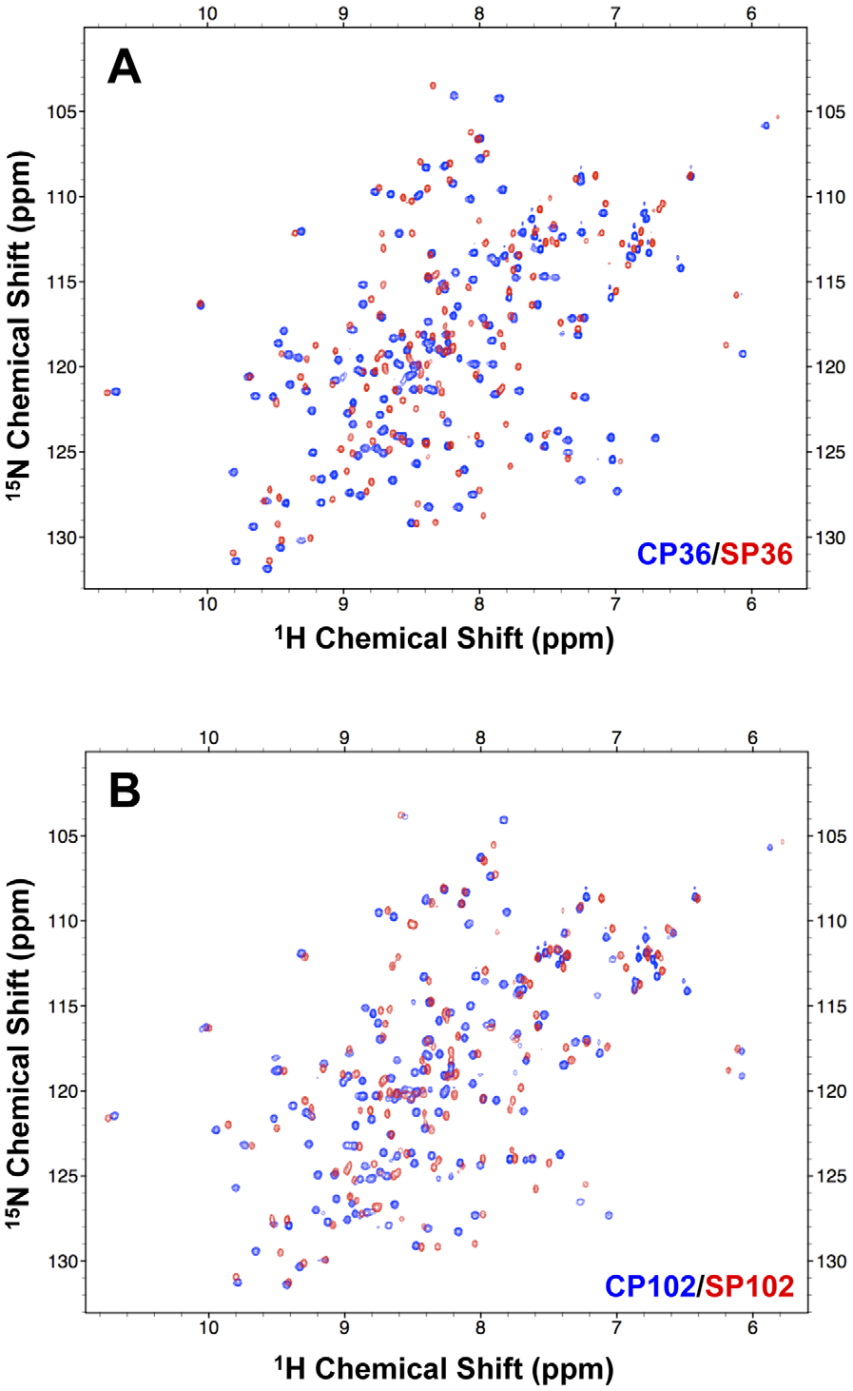
Superposition of the ^1^H-^15^N HSQC of NpuInt SP and the corresponding CP variant showing the overall correspondence between two spectra. (A) NpuInt CP36 (blue) and NpuInt SP36 (red). (B) NpuInt CP102 (blue) and NpuInt SP102 (red).

**Figure 5:**
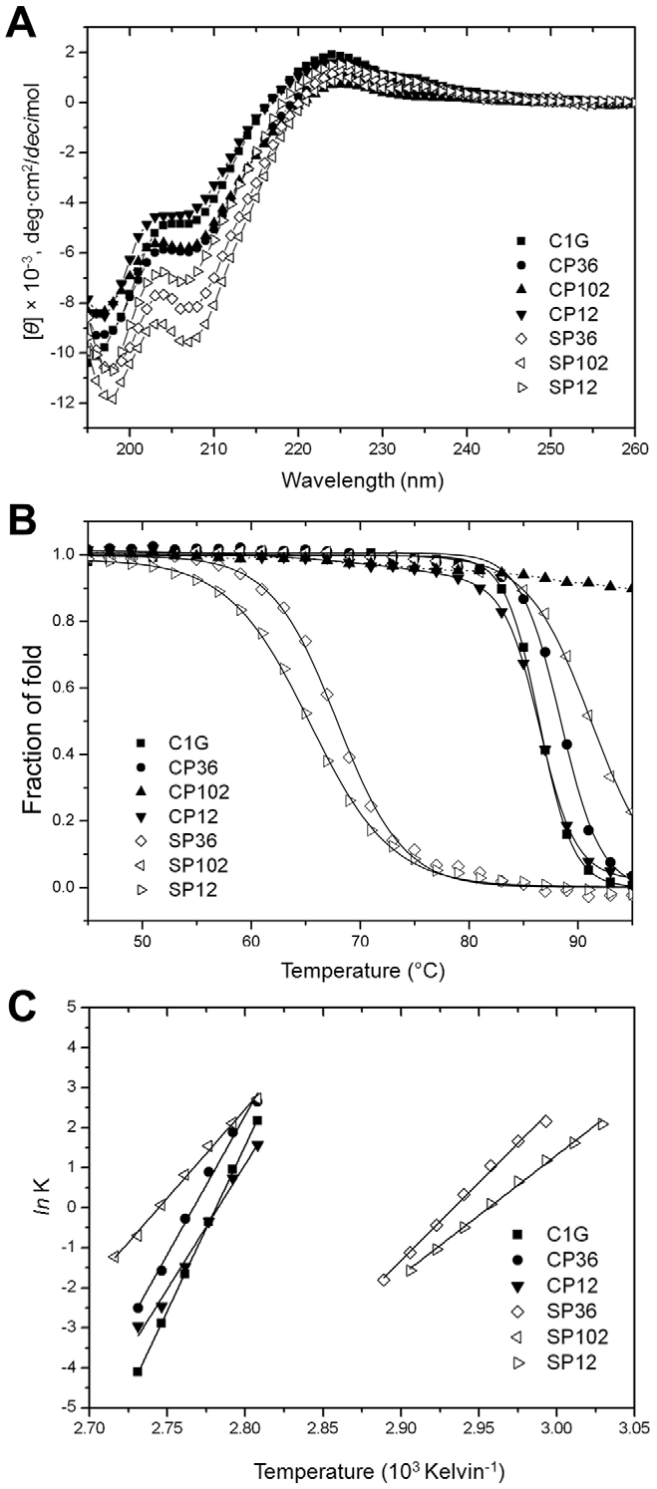
Thermal stability of intein variants monitored by CD ellipticity at 224 nm. (A) Far-UV CD spectra of NpuInt variants at 25°C. (B) The two-state thermal denaturation profiles of NpuInt variants. (C) Temperature dependence of the denaturation thermodynamics using van’t Hoff analysis.

To evaluate the feasibility of converting a CP site to a split site, we created split NpuInts in which a backbone opening was introduced at the identified CP sites (residues 12, 36 and 102) and designated them SP12 (SP12^N^/SP12^C^), SP36 (SP36^N^/SP36^C^) and SP102 (SP102^N^/SP102^C^). The ^1^H-^15^N HSQCs of the two-fragment constructs showed that the molecules are also well structured, resulting in similarly distributed cross peaks as observed in the corresponding CP variants (Figure 4 and S1). The introducing of the split sites was not structurally detrimental in the SP constructs. We subjected these SPs, along with CPs, to CD measurement to evaluate their structural properties. The NpuInt CPs and SPs were very similar in terms of secondary structure to the native version of NpuInt, C1G, as reflected in the CD profiles (Figure 5A). The thermal stability was further examined to define the effect of backbone opening. The CD profiles changed as a function of temperature (Figure S2), and the ellipticity at 224 nm was utilized to analyze the heat-induced denaturation of individual CPs and SPs (Figure 5B). Surprisingly, the three CPs and the native NpuInt C1G demonstrated similar substantial thermal resistance; all transition temperatures (T_M_) were higher than 85°C (Table 1). In particular, CP102 was extremely stable; the T_M_ value was too high to be precisely clarified in our measurement. As expected, two-piece split inteins demonstrated less thermal stability than the single-chain inteins (Figure 5B and Table 1). However, the SPs still showed superior thermal stability, with T_M_ >65°C, indicating well folding at ambient temperatures. SP102 has extraordinary thermal stability (T_M_ ∼91°C) in comparable to that of single-chain CPs and C1G. The thermal stability seems to be structurally associated among the various intein constructs.

**Table 1:** Thermodynamics and stability of protein unfolding of single-chain and split NpuInts.

	T_M_ (°C)	ΔH (kcal mol^−1^)	ΔS (cal mol^−1^ K^−1^)	TΔS at 298 K(kcal mol^−1^)	ΔG at 298 K (kcal mol^−1^)
CP version					
CP12	86.3	123.4±5.8	343.4±16.1	102.33	21.07
CP36	89.0	120.7±5.3	334.0±14.7	99.53	21.17
CP102	>95	–	–	–	–
SP version					
SP12	65.3	50.5±0.7	149.4±2.1	44.52	5.98
SP36	67.9	77.3±1.1	226.7±3.3	67.56	9.74
SP102	91.2	87.9±1.1	241.1±3.0	71.85	16.05
Native version					
C1G mutant	86.7	163.5±0.6	454.6±1.6	135.47	28.03

### Comparison of the Thermodynamics of the NpuInt Variants

Thermodynamic parameters of enthalpy (ΔH) and entropy (ΔS) changes between the folded and unfolded states were estimated from the temperature dependence of the equilibrium of the two states [28]. We analyzed the van’t Hoff relationship corresponding to the linear plot of ln *K versus* 1/T, which yields values for ΔS and ΔH (Figure 5C and Table 1). The unfolding of single-chain CP12 or CP36 had ΔH = 120∼123 kcal mol^−1^ and ΔS = 340 cal mol^−1^ K^−1^, while the values for CP102 could not be accurately estimated because of its extremely high T_M_. Therefore, CP12 and CP36 showed nearly identical combinations of ΔH and ΔS values, indicating similar situation in unpacking the polypeptide side chain. The native NpuInt C1G had a comparable ΔH (160 kcal mol^−1^) and a slightly higher ΔS (450 cal mol^−1^ K^−1^). The reason for this difference is unclear; however, it might be partially due to the solvent molecules coordinated in the active site in native NpuInt. Joining of the N- and C-termini in the CPs impedes the interaction between water and the active site residues located at or near the terminal ends. In the two-fragment NpuInts SP36 and SP102, unfolding had less enthalpy (ΔH = 77∼88 kcal mol^−1^) and entropy (ΔS = 226∼240 cal mol^−1^ K^−1^) than the CPs or the native form of the protein. SP12 lost even less enthalpy (ΔH = 50 kcal mol^−1^) and also had a lower increase in entropy (ΔS = 145 cal mol^−1^ K^−1^) during unfolding, possibly because the N-terminal 12-residue fragment is too short to favorably interact with its counterpart.

It is unsurprising that the additional disconnection in the SPs caused less conformational restriction, as reflected in the lower enthalpy values. However, the disconnection also introduced a higher freedom of movement in the protein backbone; thus, the entropy difference upon denaturation is somewhat reduced. The decreased enthalpy for protein folding adequately compensates for the low entropy loss during the self-association process. Therefore, the three split NpuInts gained favorable free energy for folding, and we still observed high T_M_s (Table 1). We conclude that if the backbone break is properly introduced, ΔH can be balanced by a reduction in ΔS, leaving the derived free energy ΔG negative and favoring folding in split proteins.

### In vitro Trans-splicing Assay of Split Inteins

**Figure 6:**
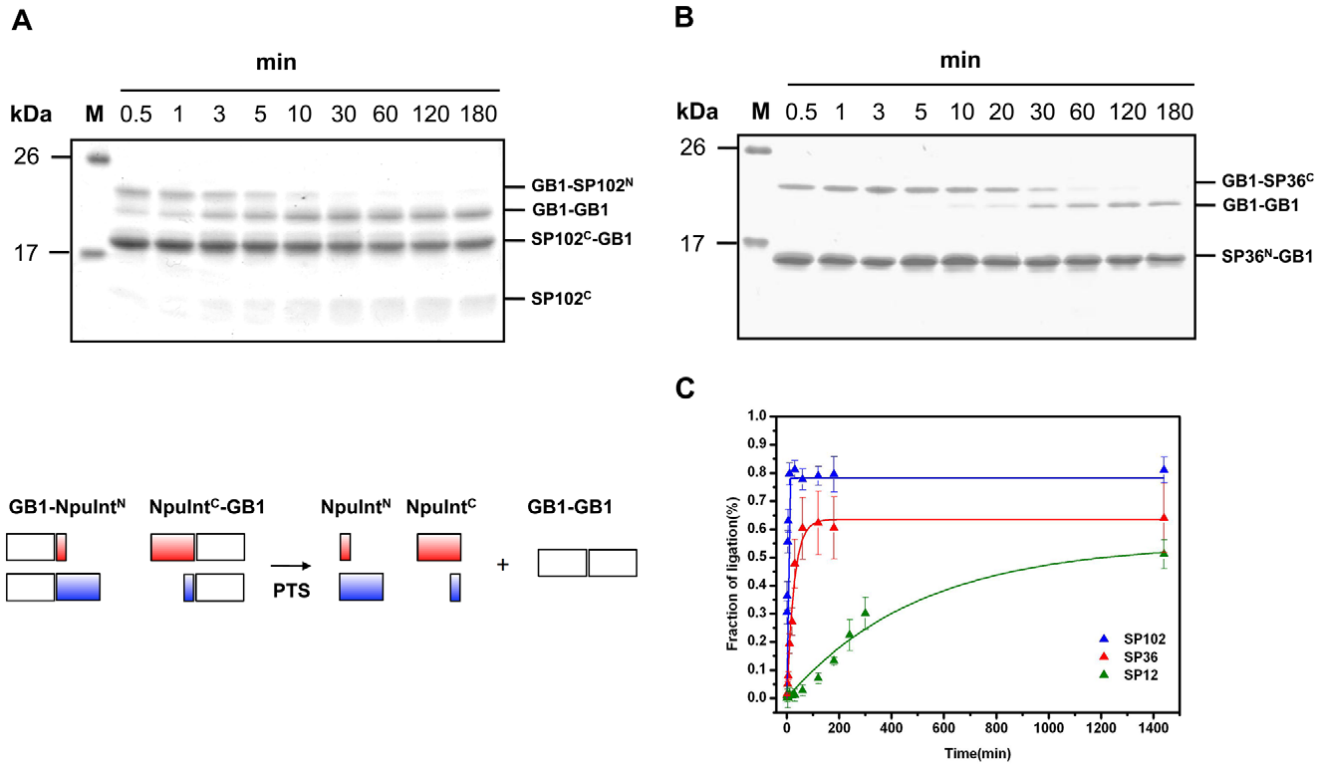
*In vitro* protein *trans*-splicing (PTS) assay. Time course of the protein ligation of GB1 and GB1 by (A) the naturally occurring split intein SP102 and (B) the engineered split intein SP36. (C) PTS kinetic analysis of the ligated product of GB1 duplication from SDS-PAGE after reaction using SP102 (blue line), SP36 (red line) and SP12 (green line). The schematic plot of the PTS reactions is depicted at the side.

We tested three folded split inteins that were disproportionally split toward the N- or C-terminus for functional PTS activity in an *in vitro* ligation assay. A strategy has been developed for robustly assaying PTS activity; following this strategy, we conjugated a well-characterized extein, GB1, to intein fragments, yielding GB1-NpuInt^N^ and NpuInt^C^-GB1 [20]. As previously reported, the ligation rate can be estimated from the production of GB1-GB1 [20], [29]. We observed the presence of significant GB1-GB1 in the GB1-SP36^N^/SP36^C^-GB1 and GB1-SP102^N^/SP102^C^-GB1 reactions (Figure 6A and B). The reaction products were confirmed by mass spectrometry (Figure S3). However, the GB1-SP12^N^/SP12^C^-GB1 reaction resulted in the formation of less GB1 (Figure S4). We next examined the individual ligation rates. The PTS rate constant of SP102 was found to be 9.0×10^−3^ s^−1^ (t_1/2_∼1.3 min), and splicing was observed to be nearly complete (80%) in 5 minutes, while the constant for SP36 was estimated to be 6.2×10^−4^ s^−1^ (t_1/2_∼18 min), and the splicing yield reached ∼65% in an hour (Figure 6C). Meanwhile, SP12 exhibited a typical PTS activity (rate constant >6.4×10^−5^ s^−1^, t_1/2_>260 min). The naturally occurring split intein, SP102, had the extremely great PTS activity and the newly identified SP36 also had very superior PTS activity comparing with other typical split inteins.

The identification of the functional split intein SP36 is remarkable. Until this work, the most feasible split inteins had split sites near the carboxyl-terminus [18]. Only one split intein with an N-terminus-proximal split site (residue 11 of *Ssp* DnaB) has been reported; this intein has moderate PTS activity (t_1/2_∼280 min, splicing yield of 48%) [18], [26]. Based on CPred prediction, we identified a novel functional split site 36 in intein, which has a short N-terminal fragment and most importantly, exhibited a significantly faster PTS rate. This split site has not been reported in other commonly used inteins.

Split inteins have been exploited in numerous protein-engineering applications, such as protein ligation, protein cyclization, and posttranslational modification through semi-synthesis. Two bottlenecks currently limit the PTS applications: one is the enzyme efficiency, and the other is the length of the short fragment. If we can create a split intein which one of the two fragments is short enough, we can chemically synthesize the short fragment. Therefore, better potential can be created in incorporating a chemical synthetic scheme into the production of the short fragment with desirable modifications on it. We demonstrated a new split intein, SP36, which has a split site disproportionally close to the N-terminus. The resulting 36 amino acid fragment is amenable to chemical synthesis. Therefore, SP36 will be useful for adding any posttranslational modification to the N-terminus of a protein of interest.

### Association between Two Pieces of Split Inteins

**Figure 7:**
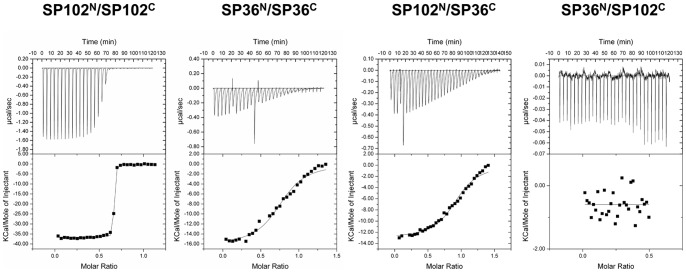
ITC measurement representing the desired specificity of the following combinations of the N- and C-termini of inteins: SP102^N^/SP102^C^, SP36^N^/SP36^C^, SP102^N^/SP36^C^ and SP36^N^/SP102^C^ (from left to right).

We suspected that the PTS efficiency might relate to the ability of fragments to associate. Therefore, we measured the binding affinity between the two complementary intein fragments of SP36 and SP102 by ITC measurement. In this assay, we used GB1-NpuInt^N^ and NpuInt^C^-GB1 in which two functionally important residues (C1 and N137) were mutated, to avoid deleterious consequences of the intein PTS reaction. The fragment pair SP102^N^ and SP102^C^ had a low-nanomolar binding affinity of 1.7 nM; SP36^N^ and SP36^C^ associated with a 700-fold weaker affinity of 1.2 µM (Figure 7). The difference of affinity is mainly derived from the change of binding enthalpy (ΔH). The association of SP102^N^/SP102^C^ had ΔH = −36.6 kcal mol^−1^ and SP36^N^/SP36^C^ had −16.6 kcal mol^−1^ while both had relatively small TΔS values in binding. Since enthalpy represents the binding energy of a thermodynamics system, we suspect that the fact might correlate with the larger binding interface in SP102^N^/SP102^C^ fragment pair to allow the better recognition. We listed the thermodynamics parameters of the ITC measurements in Table S1. We also tested the cross-association between various combinations of SP36 and SP102 fragments. Of the combinations tested, SP36^N^ showed the least *in vitro* cross-association with SP102^C^. However, the cross-association between SP36^C^ and SP102^N^ was measurable because each fragment contains a portion that is complementary to the other fragment (Figure 7).

Although the critical determinant of association is still unclear, the binding affinity difference seems to correlate with the PTS activity. The strong association between fragments enables satisfactory PTS function [22]. The outstanding PTS efficiency of the naturally occurring split intein SP102 might relate to the excellent intrinsic affinity and protein refolding ability of the two fragments.

### CP Prediction in other Split-protein Systems

**Figure 8:**
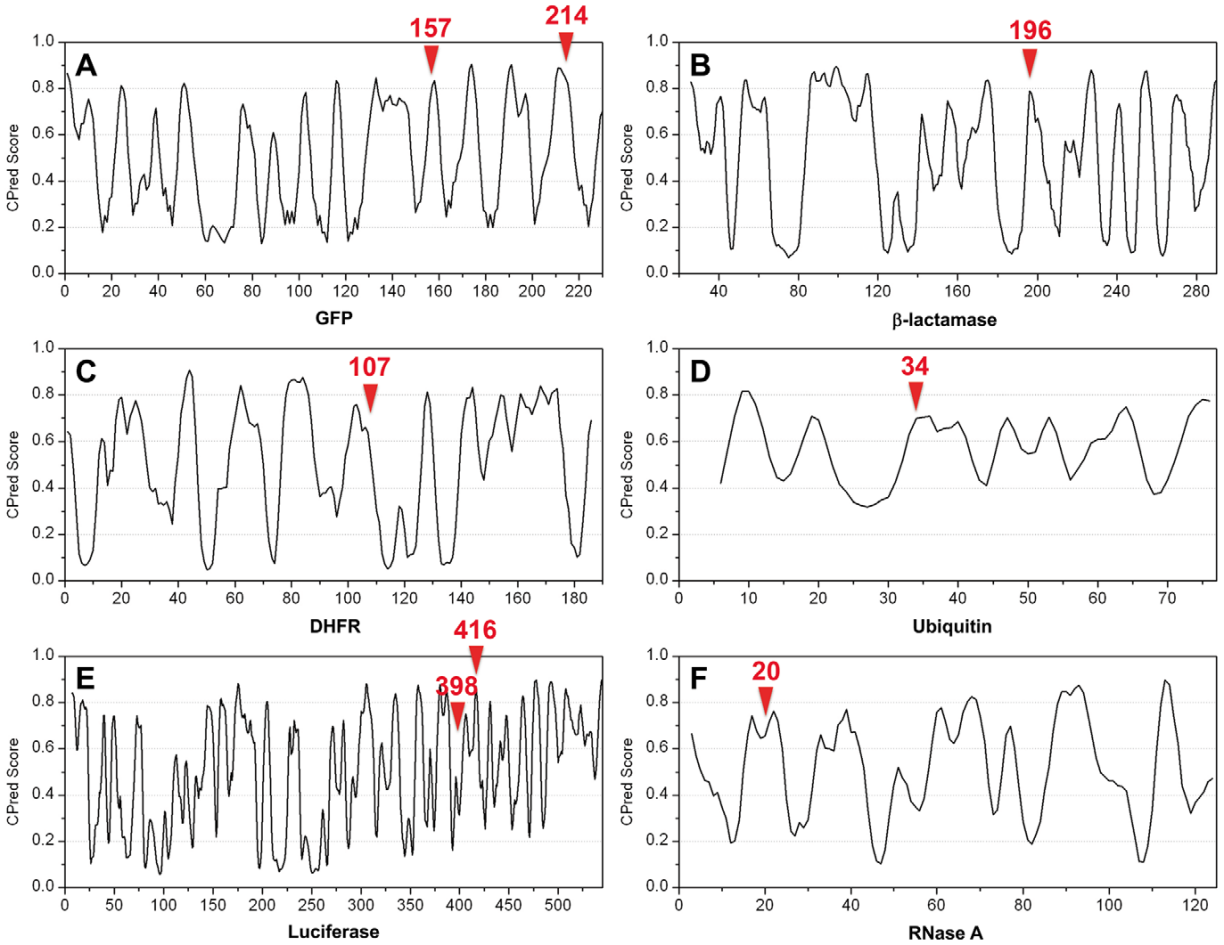
Predicted CPred profiles of different split-protein systems: (A) green fluorescence protein (GFP), (B) β-lactamase, (C) dihydrofolate reductase (DHFR), (D) ubiquitin, (E) firefly luciferase and (F) ribonuclease A (RNase A). The locations of reported split sites are indicated by red triangles.

We used our prediction system to investigate other reported split proteins to define its general applicability. Split proteins have emerged as a powerful tool for detecting biomolecular interactions and reporting biological reactions *in vivo* and *in vitro* [30], [31], [32]. There are numerous split-protein systems applied to the detection of protein-protein, protein-DNA and protein-RNA interactions, including green fluorescence protein (GFP) [30], [32], β-lactamase [31], [33], dihydrofolate reductase (DHFR) [34], ubiquitin [35], luciferase [36], [37] and ribonuclease (RNase) [38]. The reported split sites have self-assembling features. We submitted the corresponding protein structures to CPred. We found a high level of consistency, in that almost all reported split sites were close to preferred CP sites (Table 2). All of the preferred CP sites had satisfactory CPred scores (>0.7). The only exception was M398 of firefly luciferase [36] for which we could not identify a proximal preferred CP site. We summarized the entire CPred profiles in Figure 8. This comparison demonstrates that our approach not only supports split-intein design but also applies to other split-protein systems. Our proposed scheme to identify viable split sites has the important advantage of eliminating demanding protein constructions by focusing on only a few CP candidates.

**Table 2:** Correspondence of reported split sites and CPred scores.

Split-protein system^a^	Reported split site (CPred score)	The most proximal split site predicted by CPred (CPred score)
Green fluorescence protein (GFP)	Q157 (0.812) [32]	K158 (0.836)
	K214 (0.852) [30]	P211 (0.890)
β-lactamase	G196 (0.791) [33]	G196 (0.791)
Dihydrofolate reductase (DHFR)	N107 (0.637) [34]	P103 (0.759)
Ubiquitin	E34 (0.698) [35]	I36 (0.709)
Firefly luciferase	M398 (0.378) [31]	unidentifiable
	E416 (0.844) [31]	E417 (0.870)
Ribonuclease A (RNase)	A20 (0.656) [38]	S22 (0.763)

a The PDB coordinates used for prediction were as follows: GFP, 1GFL [45]; β-lactamase, 1BTL [46]; DHFR, 1HFR [47]; ubiquitin, 1UBQ [48]; firefly luciferase, 2D1S [49]; RNase, 1FS3 [50].

### Summary

Split-protein fragment complementation relies on interactions driven by conditional reassembly of carefully fragmented proteins. We adopted a functionally interesting protein, NpuInt, as a model to validate the idea that CP sites identified by CP prediction can be converted to split sites. Because the crucial element in determining intein function is related to the inherent intein scaffold, the evaluation of the structures of the intein CPs seems to be an appropriate criterion for proper function. Using NMR and CD measurement, we precisely evaluated these features. We concluded that our CPs and native intein shared structural similarities, based on their secondary/tertiary structures and thermodynamic features. The new backbone openings at CP sites 12, 36 and 102 had little effect on protein structure and folding. Therefore, these openings were used as split sites in the protein primary sequence. As shown above, the split inteins still preserved the native fold and enzyme function. These data, together with the correspondence observed in other split-protein systems, confirm the feasibility of our strategy and its ability to convert CP sites to SP sites. We also identified a novel functional split intein SP36 with a short N-terminal half. The current study therefore offers an efficient, systematic way to rationally design a split protein and also sheds light on the use of various fragmented inteins for protein splicing.

## Materials and Methods

### Expression and Purification of Native Intein, Intein CPs and Split Inteins

A DNA sequence corresponding to native NpuInt was chemically synthesized. The corresponding DNA products were amplified by PCR with properly designed primers. This native NpuInt construct contains a cysteine to glycine mutation (C1G) that prevents spontaneous protein splicing. The associated coding sequences for intein CPs were derived from a head-to-tail tandem-repeated DNA sequence, and the DNA concatemer served as a template for PCR amplification. We ligated the DNA sequences via the creation of two *Xho*I restriction enzyme sites introduced at the 3′- and 5′-ends of the two NpuInt sequences, followed by ligation. A three-residue (GSS) linker was added due to the incorporation of the *Xho*I site between two DNA sequences. The NpuInt CPs contain two mutations (C1G and N137A) that eliminate the splicing activity.

Another version of split protein (SP) of inteins was constructed to generate a two-piece intein that retained intrinsic splicing activity. Similar to the strategy used for NpuInt CPs, the coding sequences were amplified from head-to-tail concatemer, but in this case, six residues (CFNGSS) were introduced as a linker. Additionally, C1 and N137 were left unmutated to preserve the slicing activity. The sequence CFN has been reported to be an optimal intein splicing sequence [17], [19], [39]. Therefore, the design allowed the CFNGSS linker to be readily excised from the primary sequence, resulting in a two-piece split intein *in vivo*. All NpuInt variants were cloned into plasmid pET-6H, a modified form of pET11d (Novagen), which expresses recombinant proteins with hexa-histidine tags at the N-termini, allowing for purification. The translated sequences are all listed in Text S1.

### Protein Expression and Purification

*Escherichia coli* BL21 (DE3) cells (ECOS™ 21, Yeastern Biotech, Taipei, Taiwan) harboring a given plasmid were cultured in *Luria Bertani* (LB) media with 100 µg/ml ampicillin. Cells were cultured at 37°C until the OD600 reached 0.6 and subsequently induced by 1 mM IPTG treatment. We harvested the cells by centrifugation (6,000 *g*, 20 min) after another 16 hours of induction at 16°C. Cell pellets were resuspended in lysis buffer (10 mM sodium phosphate, pH 7.0, 150 mM NaCl, 10 mM imidazole) and disrupted using a homogenizer (GW technologies, Taipei, Taiwan). The soluble fraction containing intein protein was separated by high-speed centrifugation (30,000 *g*, 30 min). The supernatant was mixed with Ni-charged IMAC resin (GE Healthcare, Uppsala, Sweden) equilibrated with lysis buffer and separated under different concentrations of imidazole. Proteins were concentrated and further purified to homogeneity by AKTA FPLC on a Superdex 75 size-exclusion column (GE Healthcare). The procedure was applied to all of the NpuInt variants because of their similar behavior in solution.

We observed that the CFNGSS peptide linker in the split inteins was cleaved from the expressed products during the process of *in vivo* expression. The N- and C-fragments of split intein (NpuInt^N^ and NpuInt^C^) could be copurified and detected by sodium dodecyl sulfate polyacrylamide gel electrophoresis (SDS-PAGE). We validated the correct molecular weight by mass spectroscopy.

### NMR Backbone Resonance Assignment

For NMR analysis, NpuInt CPs and native intein (C1G) were expressed in M9 medium supplemented with ^15^NH_4_Cl and ^13^C-glucose as the sole nitrogen and carbon sources. After purification, protein samples were exchanged into NMR buffer (10 mM sodium phosphate buffer, pH 6.5, 150 mM sodium chloride, 50 mM dithiothreitol (DTT), 10% D_2_O). The final samples were concentrated to 1 mM and transferred into Shigemi NMR tubes. NMR measurements were performed on Bruker 500 MHz or 600 MHz spectrometers. For backbone resonance assignments, ^1^H-^15^N HSQC [40], HNCA [41], HN(CO)CA [41], HN(CA)CB [42] and HN(COCA)CB [42] experiments were carried out at 298K. The acquired spectra were processed using NMRpipe [43] and analyzed using Sparky [44].

### Circular Dichroism (CD) Measurements

For CD experiments, samples containing 15 µM protein CD buffer (10 mM sodium phosphate buffer, pH 7.0) were used. CD spectra were recorded on an AVIV 62A DS spectropolarimeter (Lakewood, NJ, USA) from 260 to 195 nm. Triplicate scans were obtained, with an average of five repeats. The transition temperature (T_M_) of heat-induced unfolding was measured by observing the change in ellipticity as a function of temperature at 224 nm. To understand the thermodynamics of protein unfolding, the enthalpy (ΔH) and entropy (ΔS) differences were estimated from the temperature dependence of the population of folded and denatured states using the van’t Hoff relationship ln *K* = − ΔH/RT + ΔS/R. In this equation, the equilibrium constant *K* is determined using the equation *K* = [F]/[D] in which [F] and [D] represent the concentrations of the folded and denatured states, respectively, at any given temperature [28]. A linear plot of ln *K versus* 1/T will yield a y-intercept of ΔS/R and a slope of −ΔH/R.

### Preparation of Split Inteins for the Trans-splicing Assay

*Streptococcal* protein GB1 domain (GB1) was conjugated to the NpuInt^N^ or NpuInt^C^ for use as a PTS substrate [19], [20]. For GB1-NpuInt^N^ fragments, NpuInt^N^ coding sequences were amplified and subcloned into the expression vector pET-28a (Novagen), and the GB1 sequence was inserted in front of the NpuInt^N^ sequences to create GB1-NpuInt^N^. We employed the same strategy to generate NpuInt^C^-GB1. All translated GB1-coupled split intein fragments are listed in the Text S1. Protein expression and purification was routinely performed as described above, except that the cells were cultured in the presence of 50 µg/ml kanamycin. The samples were subsequently exchanged into reaction buffer (10 mM sodium phosphate buffer, pH 7.0, 0.5 mM Tris (2-carboxyethyl) phosphine (TECP) and 150 mM sodium chloride) for the PTS assay.

### In vitro Trans-splicing Assay

To examine the *trans*-splicing activity of various split inteins, GB1-NpuInt^N^ was mixed with NpuInt^C^-GB1 in reaction buffer and the reactions incubated at 25°C with vigorous shaking. Samples were collected at various time points, and the individual reactions were stopped by adding an equal amount of 2X SDS sample dye and heating to 95°C for 5 minutes. We analyzed the samples on 15% glycine-Tris SDS-PAGE gels. The ligation yield was estimated from the intensities of the bands of ligated product of GB1-GB1, which were quantitated by gel scanning using ScanMaker 9800XL (Microtek, Hsinchu, Taiwan). Because the protein *trans*-splicing reaction is assumed to be a pseudo-first-order reaction, the kinetics are described by the exponential equation A = A_0_ (1−e^-*k*t^), where *k* represents the PTS rate constant [20], [29]. An excess of short fragment is mixed with the complementary long fragment to ensure that the PTS reaction approaches first-order kinetics. The kinetic curves represent the global fit of three replicate data sets.

### Isothermal Titration Calorimetry (ITC)

Deficient GB1-NpuInt^N^ and NpuInt^C^-GB1 (with mutations of C1G and N137A) were used for ITC measurement, which was performed via VP-ITC calorimetry (GE Healthcare). A working cell of 1.4 mL in volume was filled with the long fragment of split intein at a concentration of 200−400 µM (depending on the self-association affinity). We stepwise titrated the complementary short fragment: 30 aliquots of 5-µl protein solution (20−30 µM) were injected into the working cell. Experiments were performed at 25°C in 50 mM phosphate buffer (pH 7.0), 1 mM EDTA, 300 mM NaCl and 50 mM DTT. The working cell was constantly stirred at a rate of 290 rpm during the period of measurement. The protein concentrations were determined by analyzing the sample absorption using Nanophotometer™ Pearl (IMPLEN, Munchen, Germany). All samples were degassed for 15 min before the ITC experiment to avoid the presence of bubbles.

## Supporting Information

Figure S1 ^1^H-^15^N HSQCs for one-fragment NpuInts (A)-(D) and two-fragment NpuInts (E)-(F): (A) Native NpuInt C1G, (B) NpuInt CP12, (C) NpuInt CP36, (D) NpuInt CP102, (E) NpuInt SP36 and (F) NpuInt SP102. The spectra are all acquired at 25°C and 600 MHz.
(PDF)

Figure S2 The representative CD curves of NpuInt SP36 at different temperatures.
(PDF)

Figure S3 Mass scan of *in vitro* PTS reaction of the combination of GB1-SP36^N^/SP36^C^-GB1. Protein ligation product of H_6_-GB1-GB1-H_6_ is indicated by arrow. The measured and theoretical molecular weights (MW) are labeled on the top of the individual peaks and the differences are less than 200 ppm.
(PDF)

Figure S4 Time course of *in vitro* protein *trans*-splicing (PTS) assay for split intein SP12.
(PDF)

Table S1 Thermodynamics parameters of ITC measurements.
(PDF)

Text S1 Translated protein sequences.
(PDF)
