# Supplementary material for: Circular Permutation Prediction Reveals a Viable Backbone Disconnection for Split Proteins: An Approach in Identifying a New Functional Split Intein
Source: PLoS One. 2012 Aug 24;7(8):e43820. doi: 10.1371/journal.pone.0043820 (PMC3427171; doi:10.1371/journal.pone.0043820)
Supplement: Figure S1 — 1H-15N HSQCs for one-fragment NpuInts (A)-(D) and two-fragment NpuInts (E)-(F): (A) Native NpuInt C1G, (B) NpuInt CP12, (C) NpuInt CP36, (D) NpuInt CP102, (E) NpuInt SP36 and (F) NpuInt SP102. The spectra are all acquired at 25°C and 600 MHz. (PDF) [file pone.0043820.s001.pdf]

Supporting Figure S1

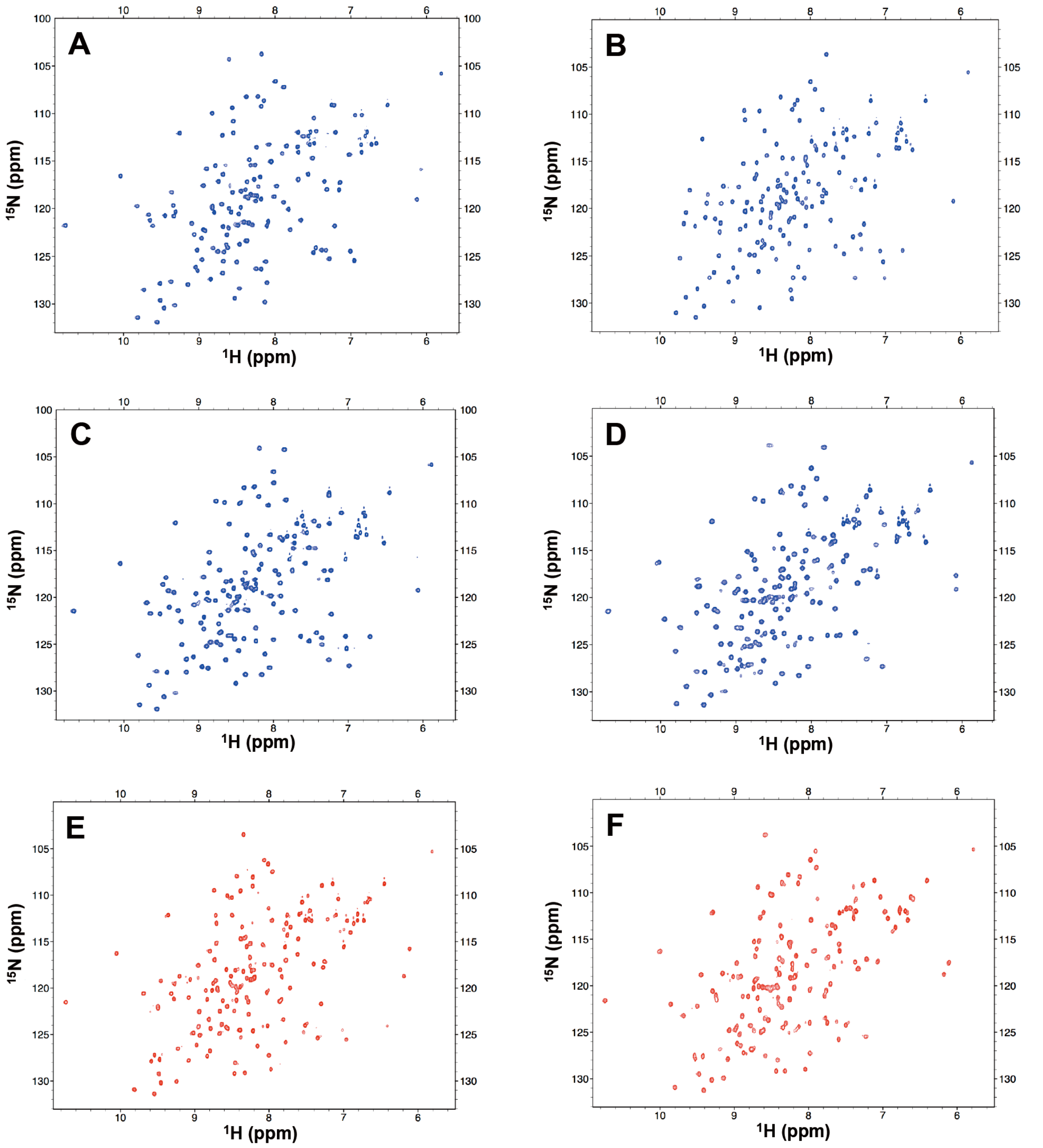

Figure S1. ^{1}H-^{15}N HSQCs for one-fragment NpuInts (A)-(D) and two-fragment NpuInts (E)-(F): (A) Native NpuInt C1G, (B) NpuInt CP12, (C) NpuInt CP36, (D) NpuInt CP102, (E) NpuInt SP36 and (F) NpuInt SP102. The spectra are all acquired at 25°C and 600 MHz.
