# Supplementary material for: Circular Permutation Prediction Reveals a Viable Backbone Disconnection for Split Proteins: An Approach in Identifying a New Functional Split Intein
Source: PLoS One. 2012 Aug 24;7(8):e43820. doi: 10.1371/journal.pone.0043820 (PMC3427171; doi:10.1371/journal.pone.0043820)
Supplement: Figure S2 — The representative CD curves of NpuInt SP36 at different temperatures. (PDF) [file pone.0043820.s002.pdf]

Supporting Figure S2

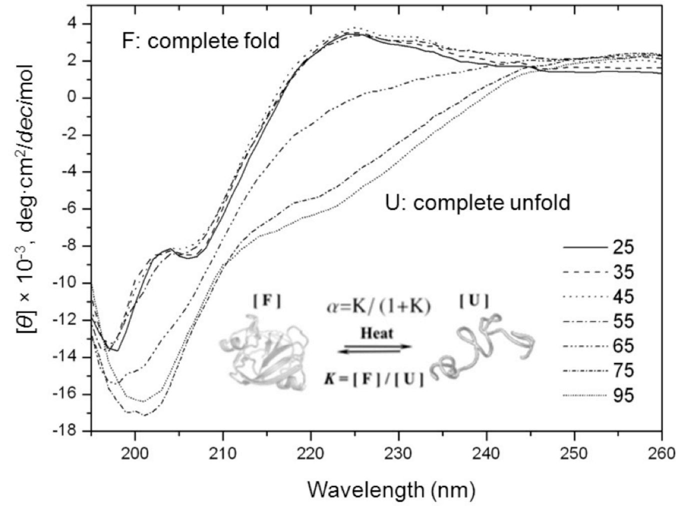

Figure S2. The representative CD curves of NpuInt SP36 at different temperatures.
