# Supplementary material for: Circular Permutation Prediction Reveals a Viable Backbone Disconnection for Split Proteins: An Approach in Identifying a New Functional Split Intein
Source: PLoS One. 2012 Aug 24;7(8):e43820. doi: 10.1371/journal.pone.0043820 (PMC3427171; doi:10.1371/journal.pone.0043820)
Supplement: Figure S3 — Mass scan of in vitro PTS reaction of the combination of GB1-SP36N/SP36C-GB1. Protein ligation product of H6-GB1-GB1-H6 is indicated by arrow. The measured and theoretical molecular weights (MW) are labeled on the top of the individual peaks and the differences are less than 200 ppm. (PDF) [file pone.0043820.s003.pdf]

Supporting Figure S3

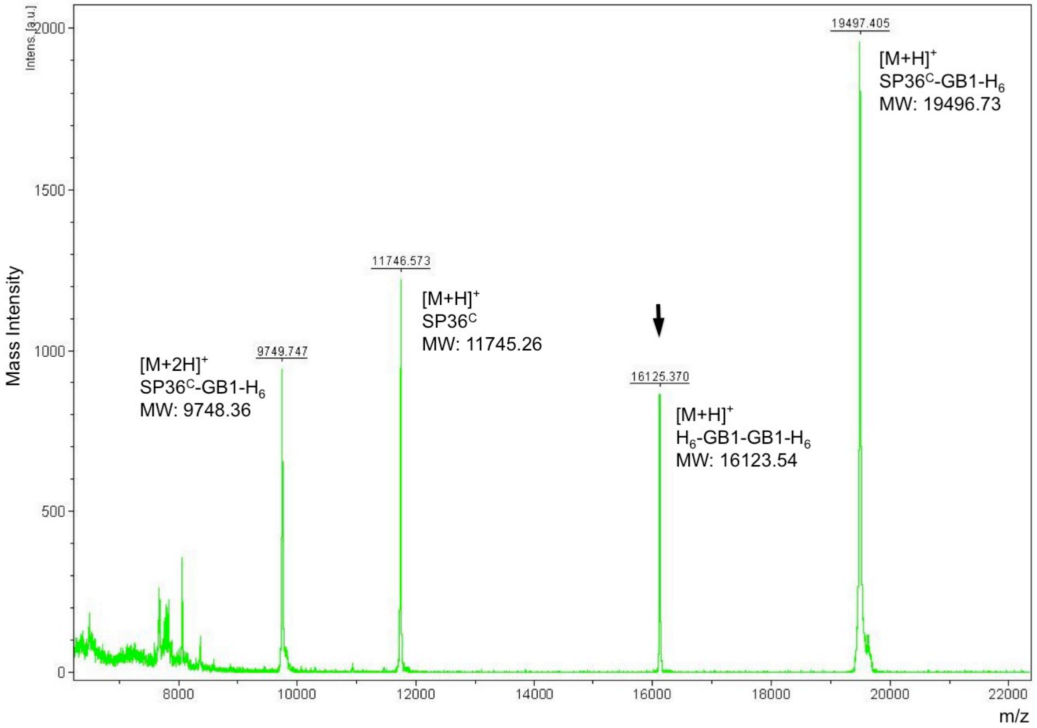

Figure S3. Mass scan of *in vitro* PTS reaction of the combination of GB1-SP36^{N}/SP36^{C}-GB1. Protein ligation product of H_6-GB1-GB1-H_6 is indicated by arrow. The measured and theoretical molecular weights (MW) are labeled on the top of the individual peaks and the differences are less than 200 ppm.
