# Supplementary material for: Circular Permutation Prediction Reveals a Viable Backbone Disconnection for Split Proteins: An Approach in Identifying a New Functional Split Intein
Source: PLoS One. 2012 Aug 24;7(8):e43820. doi: 10.1371/journal.pone.0043820 (PMC3427171; doi:10.1371/journal.pone.0043820)
Supplement: Figure S4 — Time course of in vitro protein trans-splicing (PTS) assay for split intein SP12. (PDF) [file pone.0043820.s004.pdf]

**Supporting Figure S4**


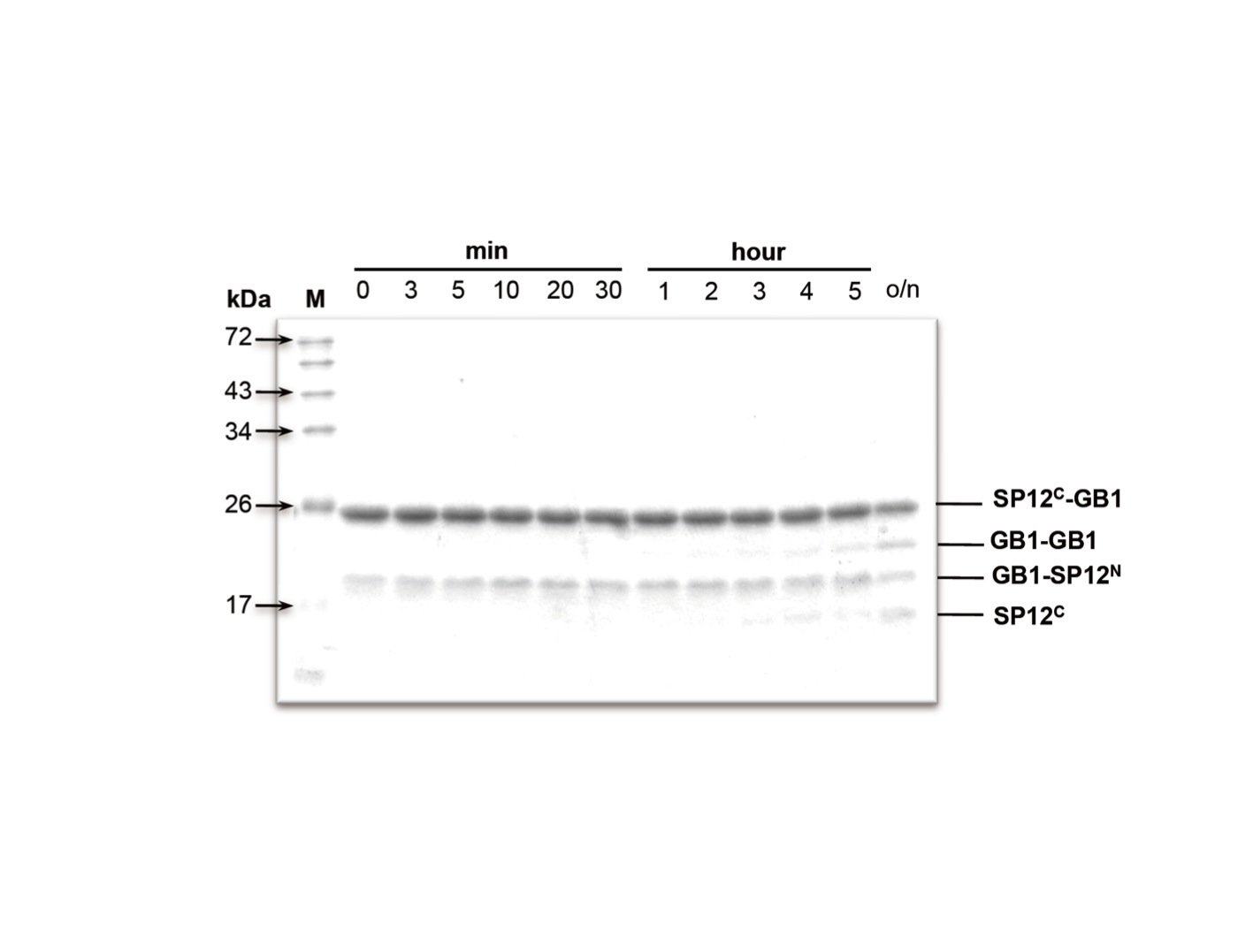


Figure S4. Time course of *in vitro* protein *trans*-splicing (PTS) assay for split intein SP12.
