# Supplementary material for: Circular Permutation Prediction Reveals a Viable Backbone Disconnection for Split Proteins: An Approach in Identifying a New Functional Split Intein
Source: PLoS One. 2012 Aug 24;7(8):e43820. doi: 10.1371/journal.pone.0043820 (PMC3427171; doi:10.1371/journal.pone.0043820)
Supplement: Table S1 — Thermodynamics parameters of ITC measurements. (PDF) [file pone.0043820.s005.pdf]

Supporting Table S1

Table S1. Thermodynamics parameters of ITC measurements.

Protein (In Cell)	Protein (In syringe)	N	K_A (10^6 M^{-1})	K_D (nM)	ΔG (kcal/mol)	ΔH (kcal/mol)	TΔS (kcal/mol)
$SP102^N$	$SP102^C$	0.65 ± 0.01	597 ± 133	1.76 ± 0.39	− 34.6	− 36.6	− 2.0
$SP36^C$	$SP36^N$	0.78 ± 0.01	0.829 ± 0.13	1239 ± 200	− 15.5	− 16.2	− 0.7
$SP102^N$	$SP36^C$	0.93 ± 0.01	0.744 ± 0.10	1367 ± 177	− 12.6	− 13.0	− 0.4
$SP102^C$	$SP36^N$	N/A	N/A	N/A	N/A	N/A	N/A
