# Supplementary material for: Circular Permutation Prediction Reveals a Viable Backbone Disconnection for Split Proteins: An Approach in Identifying a New Functional Split Intein
Source: PLoS One. 2012 Aug 24;7(8):e43820. doi: 10.1371/journal.pone.0043820 (PMC3427171; doi:10.1371/journal.pone.0043820)
Supplement: Text S1 — Translated protein sequences. (PDF) [file pone.0043820.s006.pdf]

Supporting Text S1

TRANSLATED PROTEIN SEQUENCES

A. Constructs of one-piece and two-piece intein variants. All translated *Npu* dnaE intein (NpuInt) sequences are listed: the primary sequences of NpuInts are in ***black*** and appendage tags and linkers are in ***grey***. The mutated residues are underlined.

Native NpuInt (C1G)

```
        10         20         30         40          50         60
MHHHHHHAMG LSYETEILTV EYGLLPIGKI VEKRIECTVY SVDNNGNIYT QPVAQWHDRG
        70         80         90        100        110        120
EQEVFEYCLE DGSLIRATKD HKFMTVDGQM LPIDEIFERE LDLMRVDNLP NIKIATRKYL
       130        140
GKQNVYDIGV ERDHNFALKN GFIASN
```

NpuInt circular permutant 12 (CP12)

```
        10         20         30         40         50         60
MHHHHHHAMG YGLLPIGKIV EKRIECTVYS VDNNGNIYTQ PVAQWHDRGE QEVFEYCLED
        70         80         90        100        110        120
GSLIRATKDH KFMTVDGQML PIDEIFEREL DLMRVDNLPN IKIATRKYLG KQNVYDIGVE
       130        140        150
RDHNFALKNG FIASAGSSGL SYETEILTVE
```

NpuInt circular permutant 36 (CP36)

```
        10         20         30         40         50         60
MHHHHHHAMG GNIYTQPVAQ WHDRGEQEVF EYCLEDGSLI RATKDHKFMT VDGQMLPIDE
        70         80         90        100        110        120
IFERELDLMR VDNLPNIKIA TRKYLGKQNV YDIGVERDHN FALKNGFIAS AGSSGLSYET
       130        140        150
```

```
EILTVEYGLL PIGKIVEKRI ECTVYSVDNN
```

NpuInt circular permutant 102 (CP102)

```
        10         20         30         40         50         60
MHHHHHHAMG IKIATRKYLG KQNVYDIGVE RDHNFALKNG FIASAGSSGL SYETEILTVE
        70         80         90        100        110        120
YGLLPIGKIV EKRIECTVYS VDNNGNIYTQ PVAQWHDRGE QEVFEYCLED GSLIRATKDH
       130        140        150
KFMTVDGQML PIDEIFEREL DLMRVDNLPN
```

NpuInt split protein 12 (SP12)

```
        10         20         30         40         50         60
MHHHHHHAMG YGLLPIGKIV EKRIECTVYS VDNNGNIYTQ PVAQWHDRGE QEVFEYCLED
        70         80         90        100        110        120
GSLIRATKDH KFMTVDGQML PIDEIFEREL DLMRVDNLPN IKIATRKYLG KQNVYDIGVE
       130        140        150
RDHNFALKNG FIASNCFNGS SCLSYETEIL TVE
```

NpuInt split protein 36 (SP36)

```
        10         20         30         40         50         60
MHHHHHHAMG GNIYTQPVAQ WHDRGEQEVF EYCLEDGSLI RATKDHKFMT VDGQMLPIDE
        70         80         90        100        110        120
IFERELDLMR VDNLPNIKIA TRKYLGKQNV YDIGVERDHN FALKNGFIAS NCFNGSSCLS
       130        140        150
YETEILTVEY GLLPIGKIVE KRIECTVYSV DNN
```

NpuInt split protein 102 (SP102)

```
        10         20         30         40         50         60
```

```
MHHHHHHAMG IKIATRKYLG KQNVYDIGVE RDHNFALKNG FIASNCFNGS SCLSYETEIL
        70         80         90        100        110        120
TVEYGLLPIG KIVEKRIECT VYSVDNNGNI YTQPVAQWHD RGEQEVFEYC LEDGSLIRAT
       130        140        150
KDHKFMTVDG QMLPIDEIFE RELDLMRVDN LPN
```

B. Constructs of GB1-NpuInt^{N} and NpuInt^{C}-GB1 used in protein *trans*-splicing assay. All translated intein variants are shown in ***black*** and fusion extein GB1 and appendage tags are in ***grey***.

GB1-SP12^{N}

```
        10         20         30         40         50         60
MGSSHHHHHH SSGLVPRGSH MTYKLILNGK TLKGETTTEA VDAATAEKVF KQYANDNGVD
        70         80         90
GEWTYDDATK TFTVTEGSCL SYETEILTVE
```

SP12^{C}-GB1

```
        10         20         30         40         50         60
MGYGLLPIGK IVEKRIECTV YSVDNNGNIY TQPVAQWHDR GEQEVFEYCL EDGSLIRATK
        70         80         90        100        110        120
DHKFMTVDGQ MLPIDEIFER ELDLMRVDNL PNIKIATRKY LGKQNVYDIG VERDHNFALK
       130        140        150        160        170        180
NGFIASNCFN GSMTYKLILN GKTLKGETTT EAVDAATAEK VFKQYANDNG VDGEWTYDDA
       190
TKTFTVTELE HHHHHH
```

GB1-SP36^{N}

```
        10         20         30         40         50         60
MGSSHHHHHH SSGLVPRGSH MTYKLILNGK TLKGETTTEA VDAATAEKVF KQYANDNGVD
```

```
        70         80         90        100        110
GEWTYDDATK TFTVTEGSCL SYETEILTVE YGLLPIGKIV EKRIECTVYS VDNN
```

SP36^C-GB1

```
        10         20         30         40         50         60
MGNIYTQPVA QWHDRGEQEV FEYCLEDGSL IRATKDHKFM TVDGQMLPID EIFERELDLM
        70         80         90        100        110        120
RVDNLPNIKI ATRKYLGKQN VYDIGVERDH NFALKNGFIA SNCFNGSMTY KLILNGKTLK
       130        140        150        160        170
GETTTEAVDA ATAEKVFKQY ANDNGVDGEW TYDDATKTFT VTELEHHHHH H
```

GB1-SP102^N

```
        10         20         30         40         50         60
MGSSHHHHHH SSGLVPRGSH MTYKLILNGK TLKGETTTEA VDAATAEKVF KQYANDNGVD
        70         80         90        100        110        120
GEWTYDDATK TFTVTEGSCL SYETEILTVE YGLLPIGKIV EKRIECTVYS VDNNGNIYTQ
       130        140        150        160        170        180
PVAQWHDRGE QEVFEYCLED GSLIRATKDH KFMTVDGQML PIDEIFEREL DLMRVDNLPN
```

SP102^C-GB1

```
        10         20         30         40         50         60
MAIKIATRKY LGKQNVYDIG VERDHNFALK NGFIASNCFN GSMTYKLILN GKTLKGETTT
        70         80         90        100
EAVDAATAEK VFKQYANDNG VDGEWTYDDA TKTFTVTELE HHHHHH
```
